# Supplementary material for: SH3 interactome conserves general function over specific form
Source: Mol Syst Biol. 2013 Apr 2;9:652. doi: 10.1038/msb.2013.9 (PMC3658277; doi:10.1038/msb.2013.9)
Supplement: Supplementary Information — Supplementary Figures S1-13, Supplementary Tables S2,4,5,14 [file msb20139-s1.pdf]

SH3 Interactome Conserves General Function Over Specific Form

Xiaofeng Xin, David Gfeller, Jackie Cheng, Raffi Tonikian, Lin Sun, Ailan Guo, Lianet Lopez, Alevtina Pavlenco, Adenrele Akintobi, Yingnan Zhang, Jean-Francois Rual, Bridget Currell, Somasekar Seshagiri, Tong Hao, Xinping Yang, Yun A. Shen, Kourosh Salehi-Ashtiani, Jingjing Li, Aaron T. Cheng, Dryden Bouamalay, Adrien Lugari, David E. Hill, Mark L. Grimes, David G. Drubin, Barth D. Grant, Marc Vidal, Charles Boone, Sachdev S. Sidhu, Gary D. Bader.

Table of content:

1. Supplementary Information – Data analysis
2. Supplementary Figures 1-13
3. Supplementary Tables 1-19
4. References

1. Supplementary Information

Data analysis

Domain-protein two-way clustergram

The domain-protein two-way clustergram in Supplementary Figure 5 was generated as previously described (Jin et al, 2009), with some modifications. In particular, similarities were computed between protein-domain profiles (defined by the set of domains on each protein) and domain-protein profiles (defined by the set of proteins for each domain) using the Jaccard similarity coefficient (size of the intersection divided by the size of the union of the sets). Domain annotation was obtained from SMART (Letunic et al, 2009; Schultz et al, 2000). This

yielded statistical descriptions of the relatedness of any two proteins, based on their domain compositions, and of the relationship between any two domains based on their co-occurrence among proteins. Complete linkage hierarchical clustering was then used to cluster rows and columns of the matrix and produce a two-way clustergram of the yeast and worm SH3 protein sets. The clustergrams were generated using the MATLAB Bioinformatics Toolbox.

Compared to yeast, worm has an expanded set of domains associated with the SH3 domain, including some domains specifically present in metazoa, such as L27 domains (LIN-2 and LIN-7) which hetero-oligomerize to assemble signaling and cell polarity complexes (Harris et al, 2002), PTB (Phosphotyrosine-Binding) domains, which organize signaling complexes involved in wide-ranging physiological processes including neural development, immunity, tissue homeostasis and cell growth (Uhlik et al, 2005), and NEBU (Nebulin repeat) domains, which function in actin cytoskeleton organization and regulation (Bjorklund et al; Nakagawa et al, 2009). These observations are consistent with the "domain accretion" model proposed by Koonin et al., where protein domain organization complexity increases with organism complexity (Koonin et al, 2000).

Thematic map of the worm SH3 endocytosis interactome (Supplementary Figure 6)

To analyze whether SH3-mediated PPIs are different from other PPIs in terms of connecting proteins within or between endocytosis-related functional modules, we employed thematic map analysis using two PPI networks (Supplementary Figure 6). The first network was a sub-network of the worm SH3 interactome containing all SH3 mediated PPIs among our expert-curated worm endocytosis protein list (Supplementary Table 10). The second network contains PPIs among our endocytosis proteins retrieved from iRefWeb Release 3.2 (Turner et al, 2010), not including any SH3 proteins. The two networks were merged in Cytoscape (Shannon et al, 2003) and visualized

as a thematic map (Zhang et al, 2005) (using the Thematic Map Cytoscape plugin (Merico et al, 2011)), based on our functional annotation of worm endocytosis proteins (Supplementary Table 10). A thematic map is a simplified network view that highlights connections between modules (*e.g.*, protein biological process annotations) within an original network. In this view, endocytosis protein annotations from the original network are represented as nodes that are connected if a PPI from the original network links proteins with the corresponding annotations. *P*-values were computed by randomly shuffling the annotations of Supplementary Table 13 within the sub-networks.

2. Supplementary Figures

Figure S1

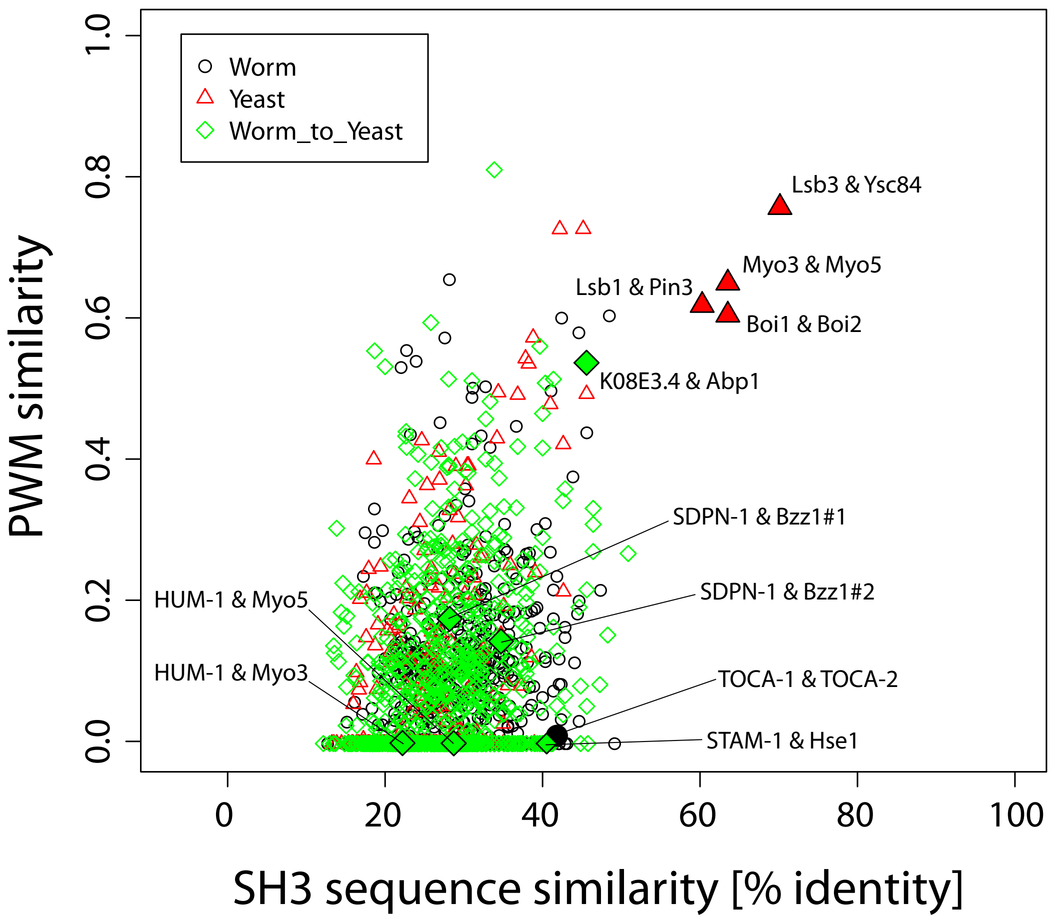

Supplementary Figure 1. Plot of the binding specificity similarity versus the sequence similarity. The sequence identity was computed based on pairwise alignments of all SH3 domains with phage display data. Binding specificity similarity is the same as the one used in the tree of Figure 1 (see Materials and Methods). For domain pairs involving domains with multiple PWMs, the highest similarity was used. Highlighted pairs correspond to domains on ortholog proteins (green) and on paralog proteins (red and black).

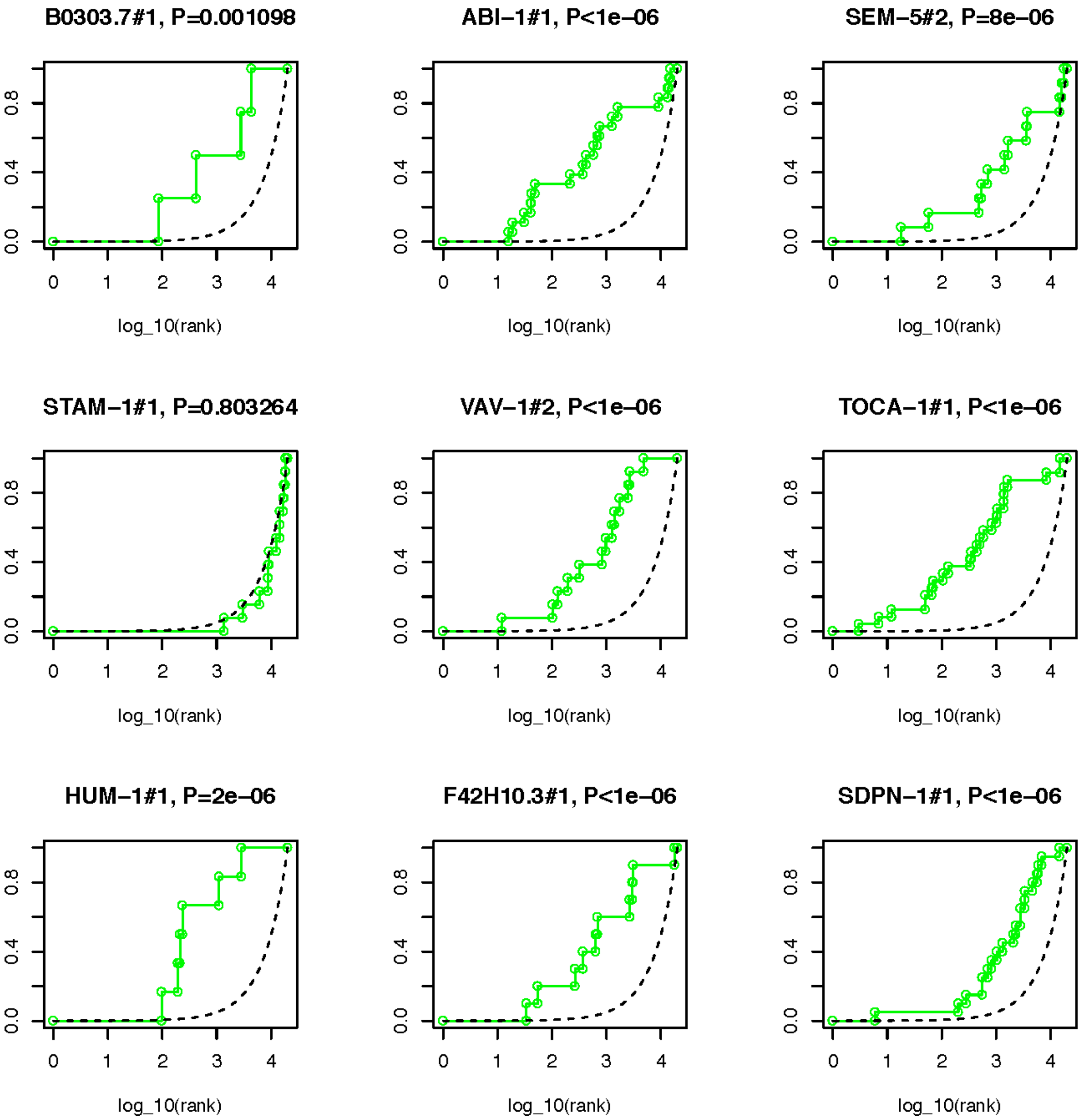
B0303.7#1, P=0.001098
ABI−1#1, P<1e−06
SEM−5#2, P=8e−06
STAM−1#1, P=0.803264
VAV−1#2, P<1e−06
TOCA−1#1, P<1e−06
HUM−1#1, P=2e−06
F42H10.3#1, P<1e−06
SDPN−1#1, P<1e−06
0.0
0.4
0.8
0
1
2
3
4
log_10(rank)

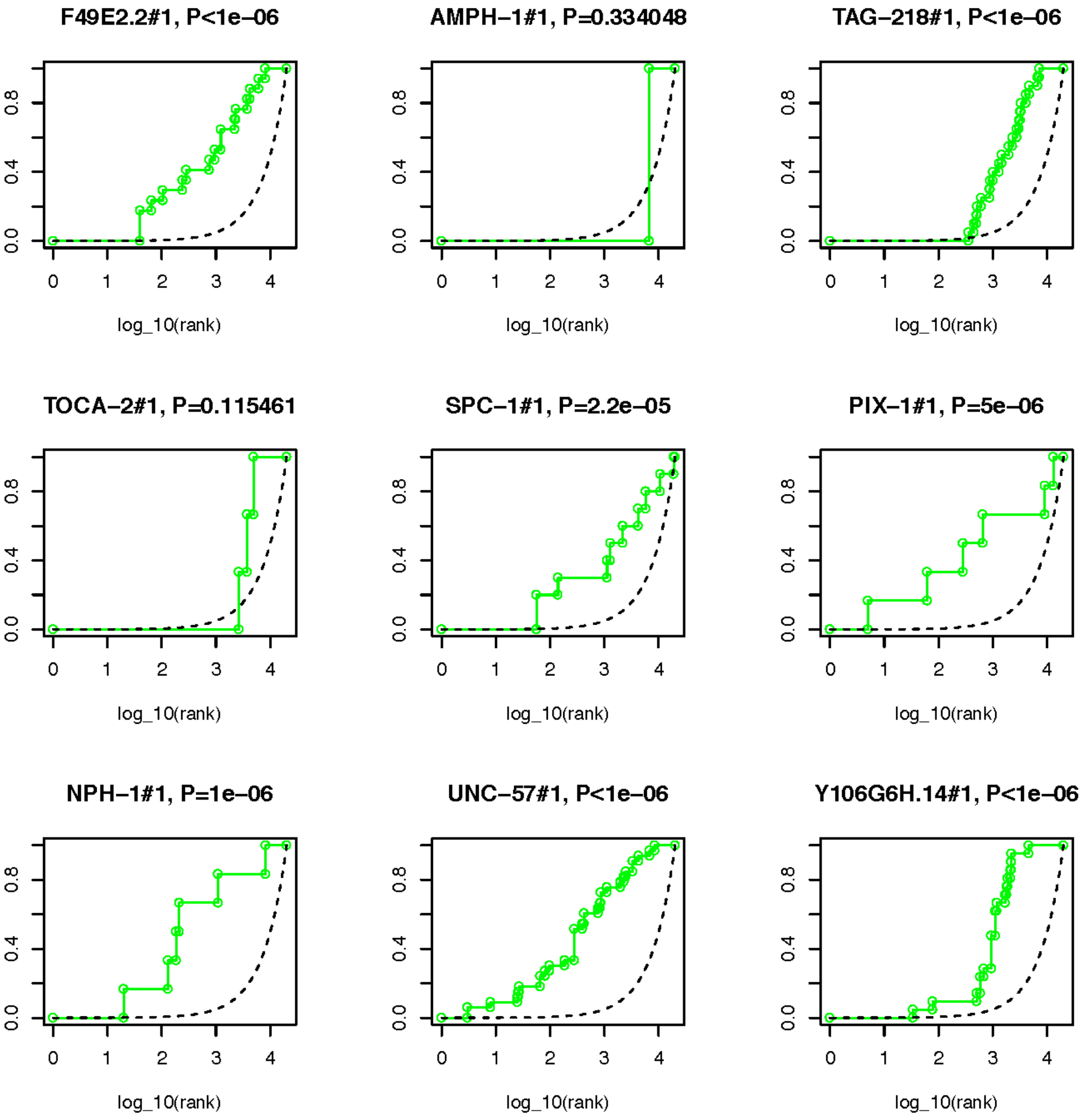
F49E2.2#1, P<1e–06
AMPH–1#1, P=0.334048
TAG–218#1, P<1e–06
TOCA–2#1, P=0.115461
SPC–1#1, P=2.2e–05
PIX–1#1, P=5e–06
NPH–1#1, P=1e–06
UNC–57#1, P<1e–06
Y106G6H.14#1, P<1e–06
0.0
0.4
0.8
0
1
2
3
4
log_10(rank)

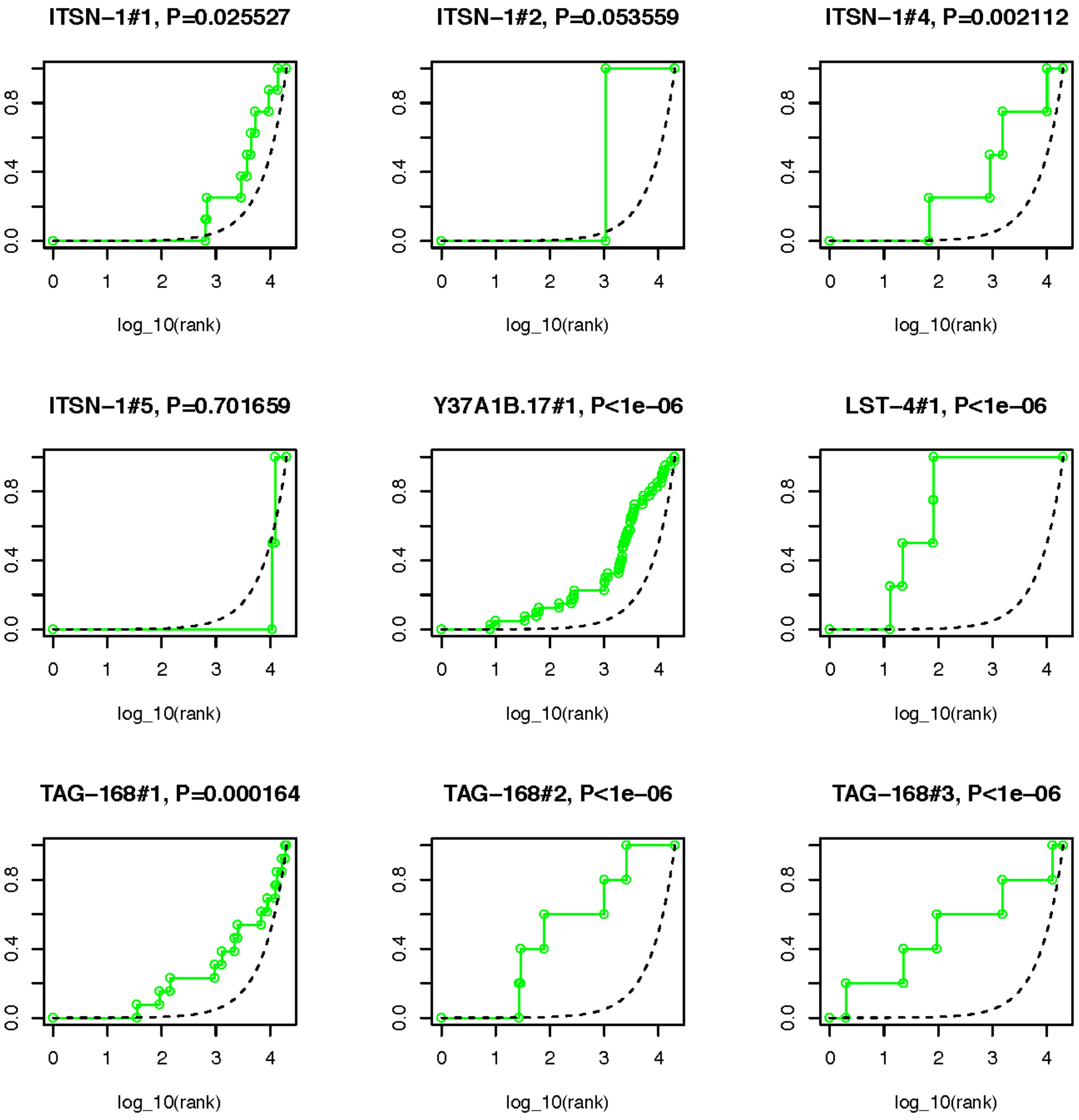
ITSN−1#1, P=0.025527
ITSN−1#2, P=0.053559
ITSN−1#4, P=0.002112
ITSN−1#5, P=0.701659
Y37A1B.17#1, P<1e−06
LST−4#1, P<1e−06
TAG−168#1, P=0.000164
TAG−168#2, P<1e−06
TAG−168#3, P<1e−06
0.0
0.4
0.8
0
1
2
3
4
log_10(rank)

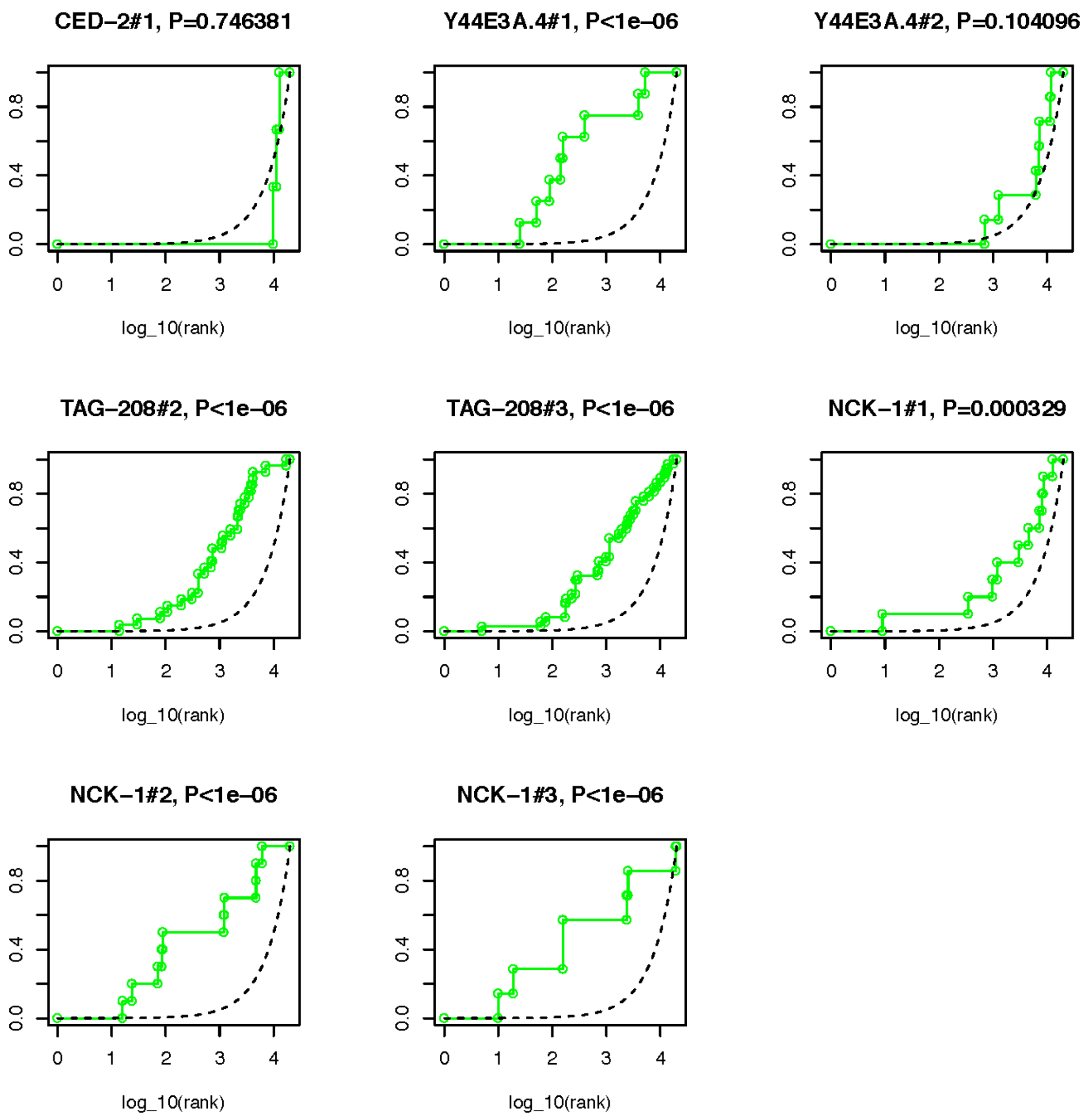

Supplementary Figure 2. Overlap between Y2H and phage display predicted PPIs for each domain. Each worm SH3 domain with a phage-derived specificity profile represented as a PWM was used to score and rank all worm proteins for matches to this PWM. The plot shows the fraction of PPIs with a rank higher than the value on the *x*-axis for each domain (green curve). The black dotted line indicates the expected distribution for random predictions.

Figure S3

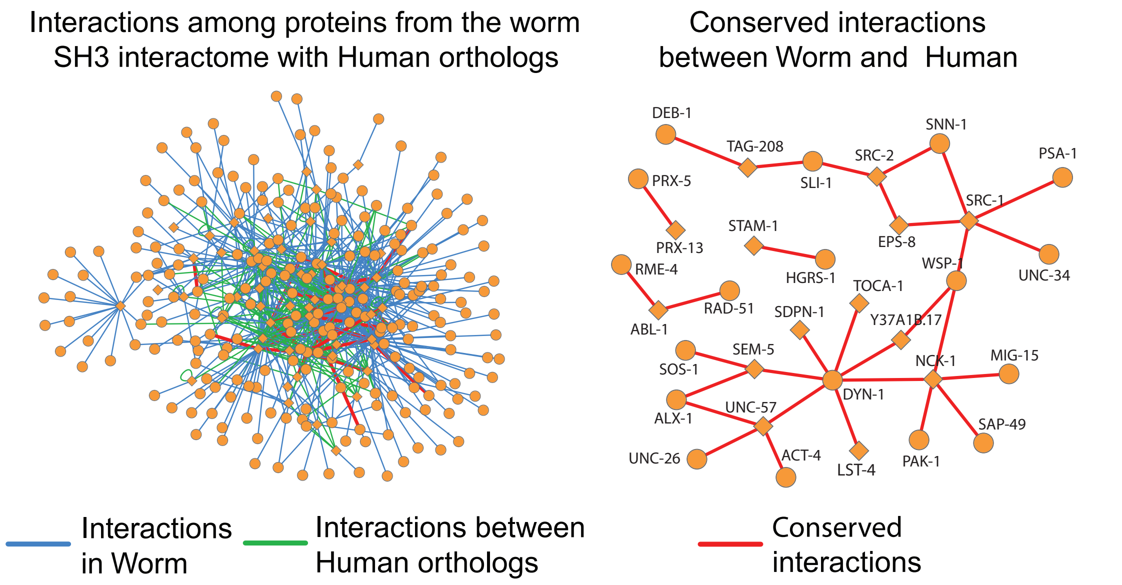

Supplementary Figure 3. Conservation of SH3-mediated protein interactions from worm to human. Worm PPIs are from our SH3 domain interactome. All human protein interactions were retrieved from the BioGRID database (Breitkreutz et al, 2008). Edges represent interactions between worm proteins (blue), human orthologs (green) and conserved across worm and human (red). Diamonds indicate SH3-containing baits.

Figure S4

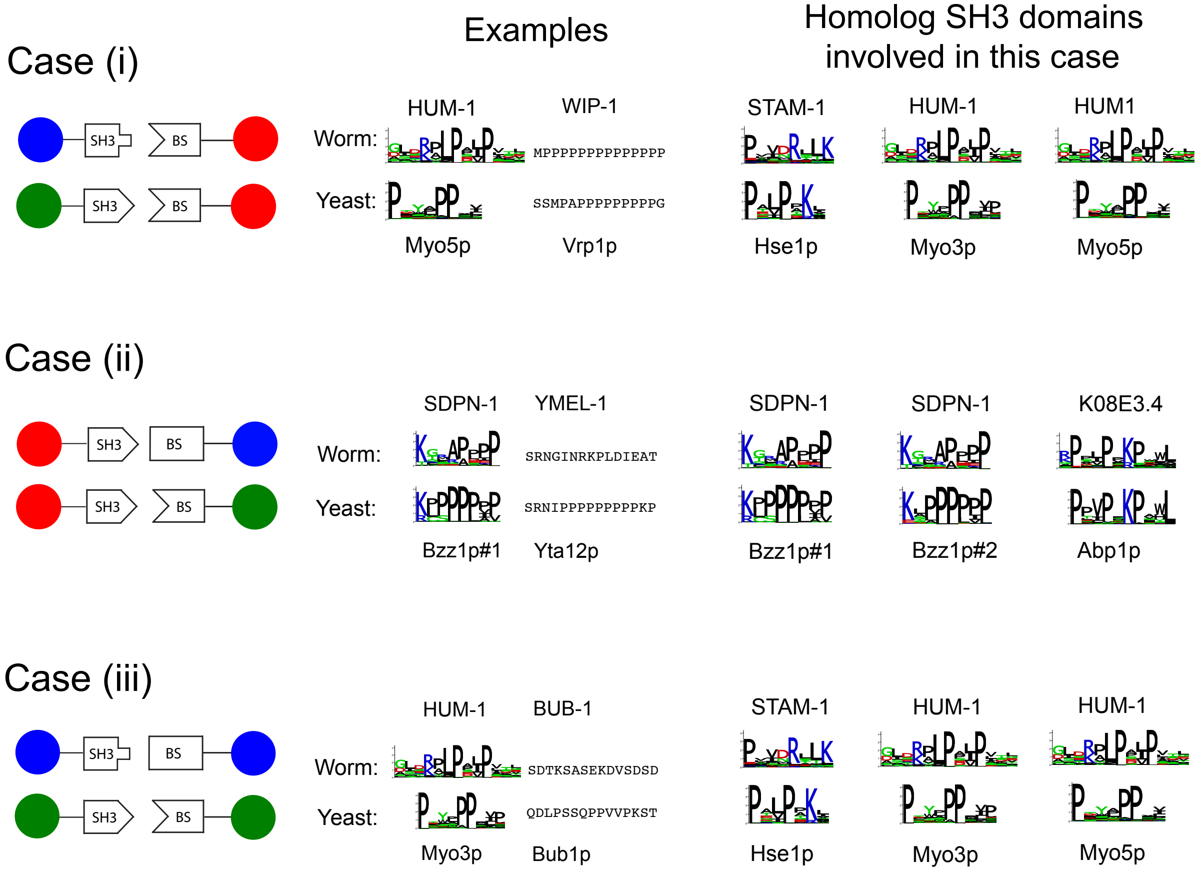

Supplementary Figure 4. Examples of rewiring occurring between worm and yeast SH3 interactomes. Case (i) corresponds to a conserved protein binding ligand but a different SH3 specificity. Case (ii) corresponds to a conserved SH3 specificity but a non-conserved binding ligand. Case (iii) corresponds to changes in both the SH3 specificity and the binding ligand. An example of each case is shown in the middle. All domains with the given rewiring case are shown at the right. Blue circles indicate worm proteins whose specificity or motif is not conserved. Green circles show yeast proteins whose specificity or motif is not conserved. Red circles show orthologs with conserved specificity or motifs.

Figure S5

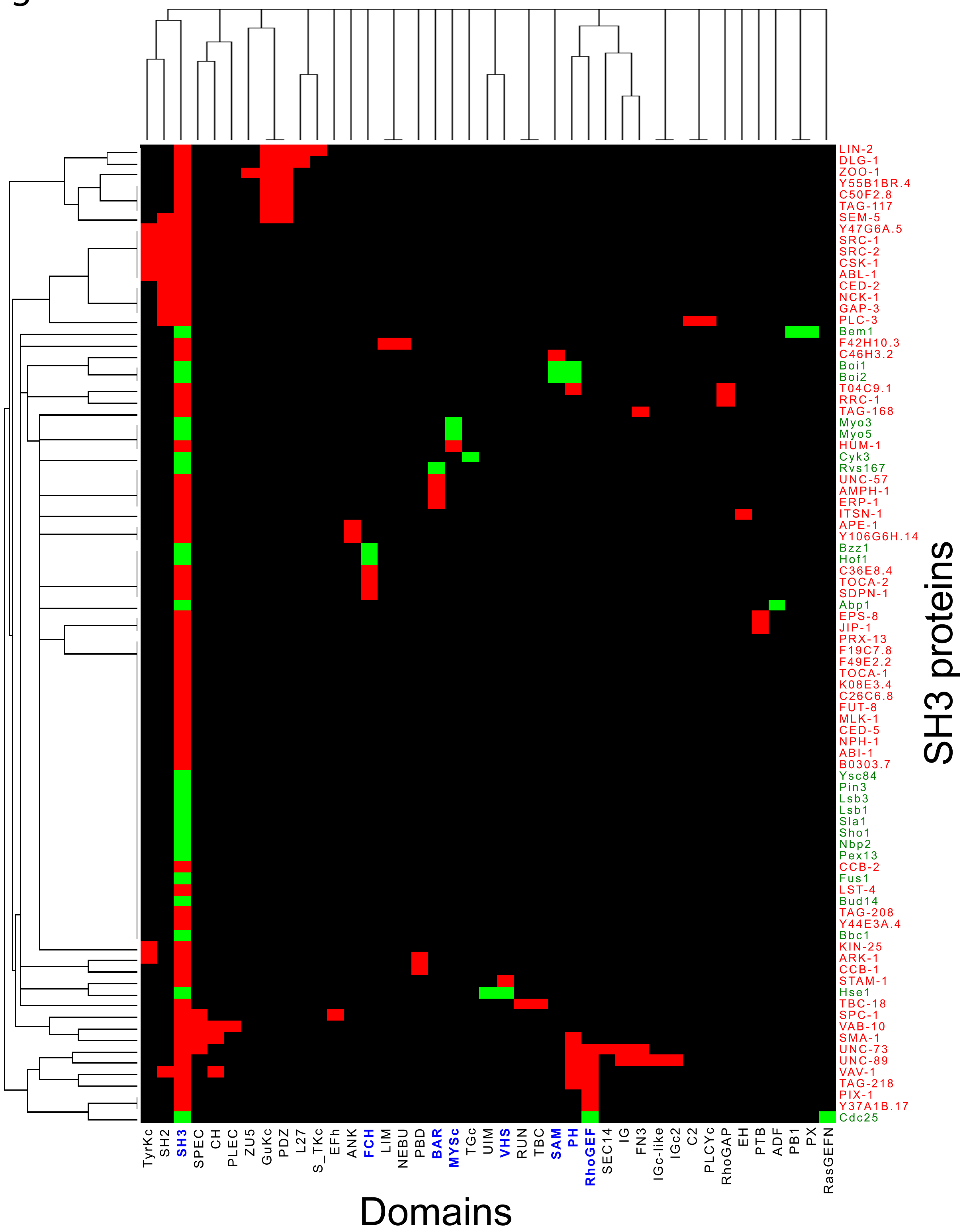

LIN-2
DLG-1
ZOO-1
Y55B1BR.4
C50F2.8
TAG-117
SEM-5
Y47G6A.5
SRC-1
SRC-2
CSK-1
ABL-1
CED-2
NCK-1
GAP-3
PLC-3
Bem1
F42H10.3
C46H3.2
Boi1
Boi2
T04C9.1
RRC-1
TAG-168
Myo3
Myo5
HUM-1
Cyk3
Rvs167
UNC-57
AMPH-1
ERP-1
ITSN-1
APE-1
Y106G6H.14
Bzz1
Hof1
C36E8.4
TOCA-2
SDPN-1
Abp1
EPS-8
JIP-1
PRX-13
F19C7.8
F49E2.2
TOCA-1
K08E3.4
C26C6.8
FUT-8
MLK-1
CED-5
NPH-1
ABI-1
B0303.7
Ysc84
Pin3
Lsb3
Lsb1
Sla1
Sho1
Nbp2
Pex13
CCB-2
Fus1
LST-4
Bud14
TAG-208
Y44E3A.4
Bbc1
KIN-25
ARK-1
CCB-1
STAM-1
Hse1
TBC-18
SPC-1
VAB-10
SMA-1
UNC-73
UNC-89
VAV-1
TAG-218
PIX-1
Y37A1B.17
Cdc25
SH3 proteins
TyrKc
SH2
SH3
SPEC
CH
PLEC
ZU5
GuKc
PDZ
L27
S_TKc
EFh
ANK
FCH
LIM
NEBU
PBD
BAR
MYSc
TGc
UIM
VHS
RUN
TBC
SAM
PH
RhoGEF
SEC14
IG
FN3
IGc-like
IGc2
C2
PLCYc
RhoGAP
EH
PTB
ADF
PB1
PX
RasGEFN
Domains

Supplementary Figure 5. Two-way clustergram showing the domain composition of yeast and worm SH3 domain containing proteins. Domains appearing in yeast SH3 proteins are represented using green blocks and domains in worm SH3 proteins are shown using red blocks. Yeast SH3 protein names are in green and worm SH3 protein names are in red. The eight domains present in both yeast and worm SH3 proteins are colored blue.

Figure S6

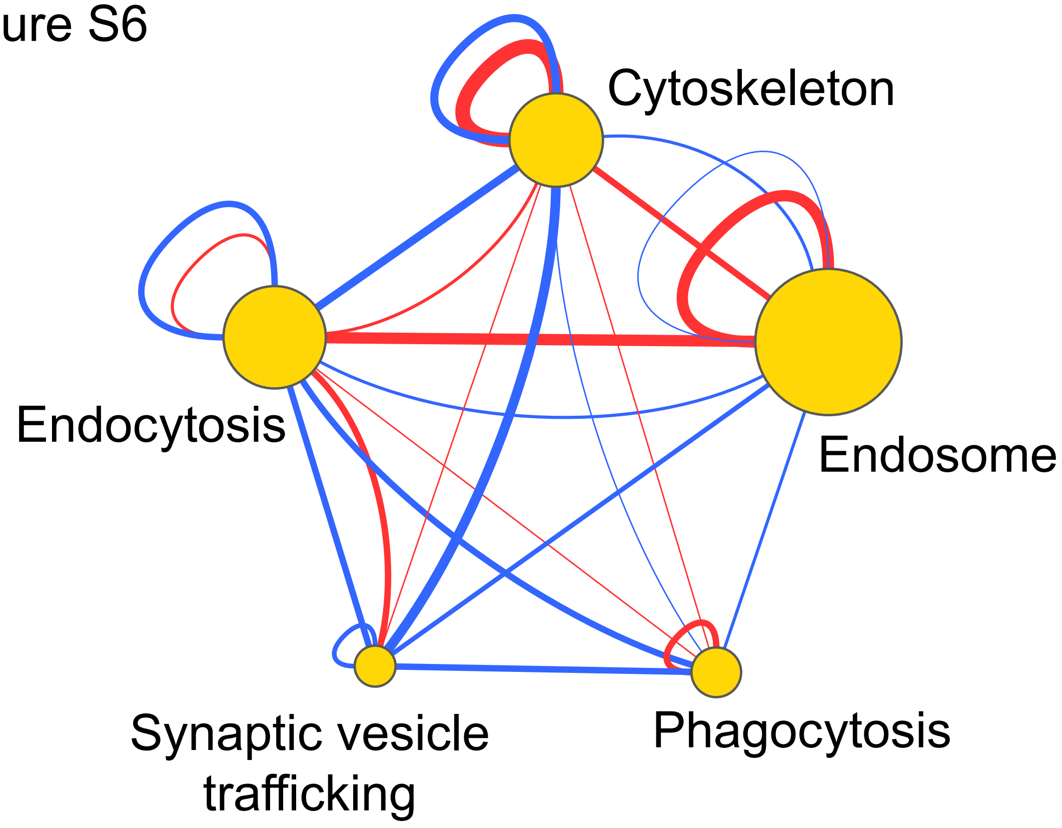

Supplementary Figure 6. Thematic map of the worm endocytosis protein interaction network. This map summarizes the PPIs within and between functional groups involved in endocytosis. Node size is proportional to the number of proteins in that group. Edge thickness is proportional to the number of PPIs between connecting groups. Edges representing SH3 domain mediated PPIs are in blue and those representing non-SH3 mediated PPIs are in red.

Figure S7

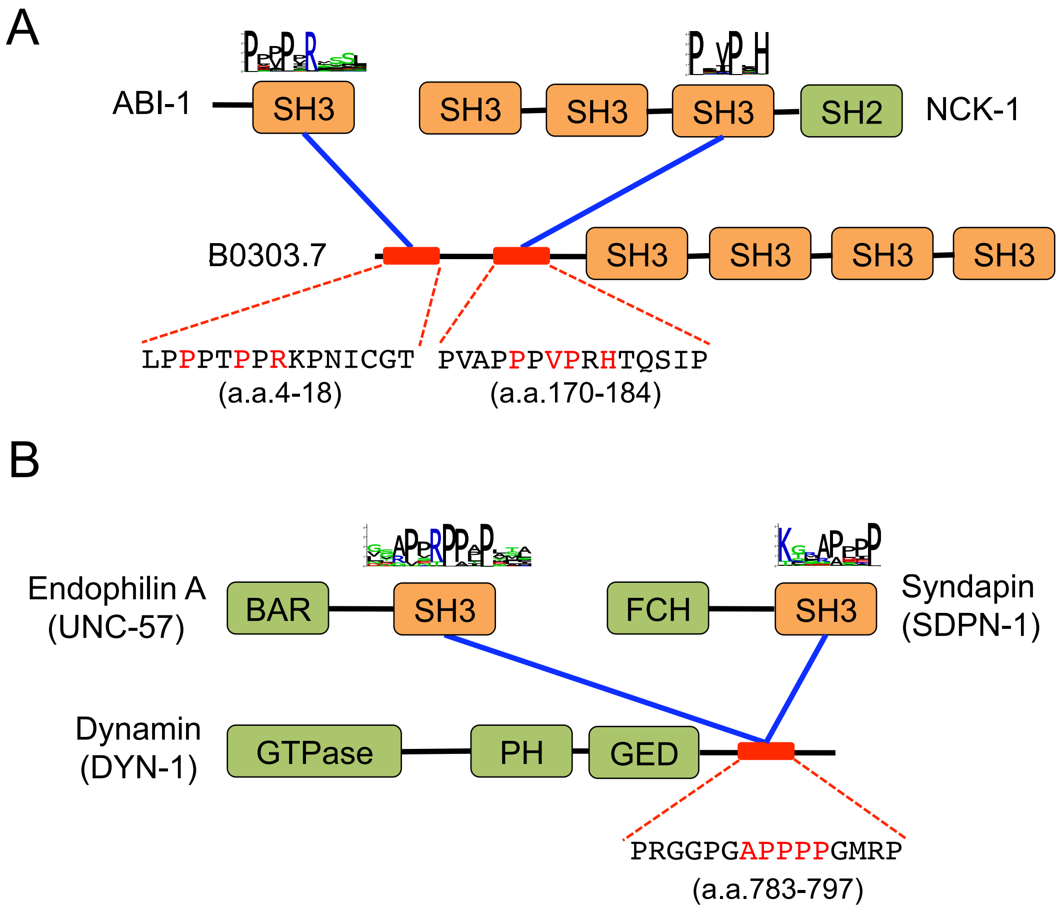

Supplementary Figure 7. Examples of coincident and competitive interactions. (A) Example of the predicted coincident interaction between the ABI-1 and the NCK-1 SH3 domains with B0303.7 at different predicted binding sites (amino acids 4-18 and 170-184, respectively). ABI-1 and NCK-1 are known to function together in regulation of actin dynamics in cell migration (Anggono & Robinson, 2007). B0303.7, with 4 SH3 domains, may function as an adaptor in the same process. (B) Example of a potentially competitive interaction between the UNC-57 (endophilin A) and the SDPN-1 (syndapin) SH3 domains, which are both predicted to bind to the same binding motif (amino acids 783-797) of DYN-1 (dynamin). All three proteins are

conserved between worm and mammals and the same competitive interactions were identified by *in vitro* protein binding assay in their rat orthologs (Anggono & Robinson, 2007).

Figure S8

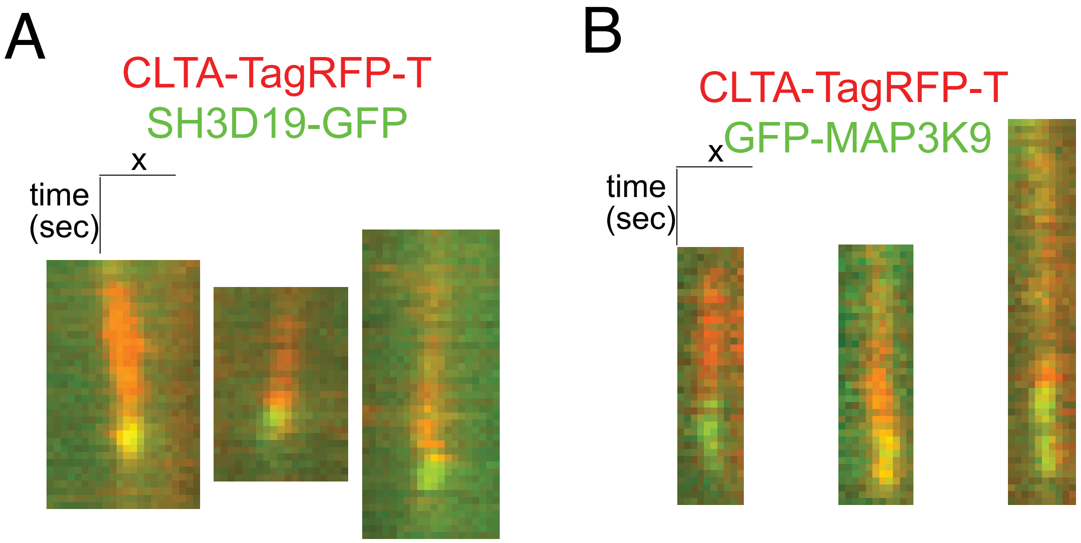

Supplementary Figure 8. Dynamics of SH3D19 and MAP3K9 localization. Dynamics of SH3D19 and MAP3K9 localization with clathrin, represented as kymographs. Time is represented on the y-axis and space on the x-axis. CLTA-TagRFP-T puncta appearing over time (red vertical shape) turn green and yellow as the GFP tagged protein localizes to the puncta, late in the appearance of the puncta. (A) Representative kymographs of individual clathrin-coated pits showing the recruitment of the SH3D19-GFP (C-terminal tag) to the CLTA-TagRFP-T puncta at the late stage of endocytosis. (B) Representative kymographs showing the recruitment of the GFP-MAP3K9 (N-terminal tag) to the CLTA-TagRFP-T puncta at the late stage of endocytosis.

Figure S9

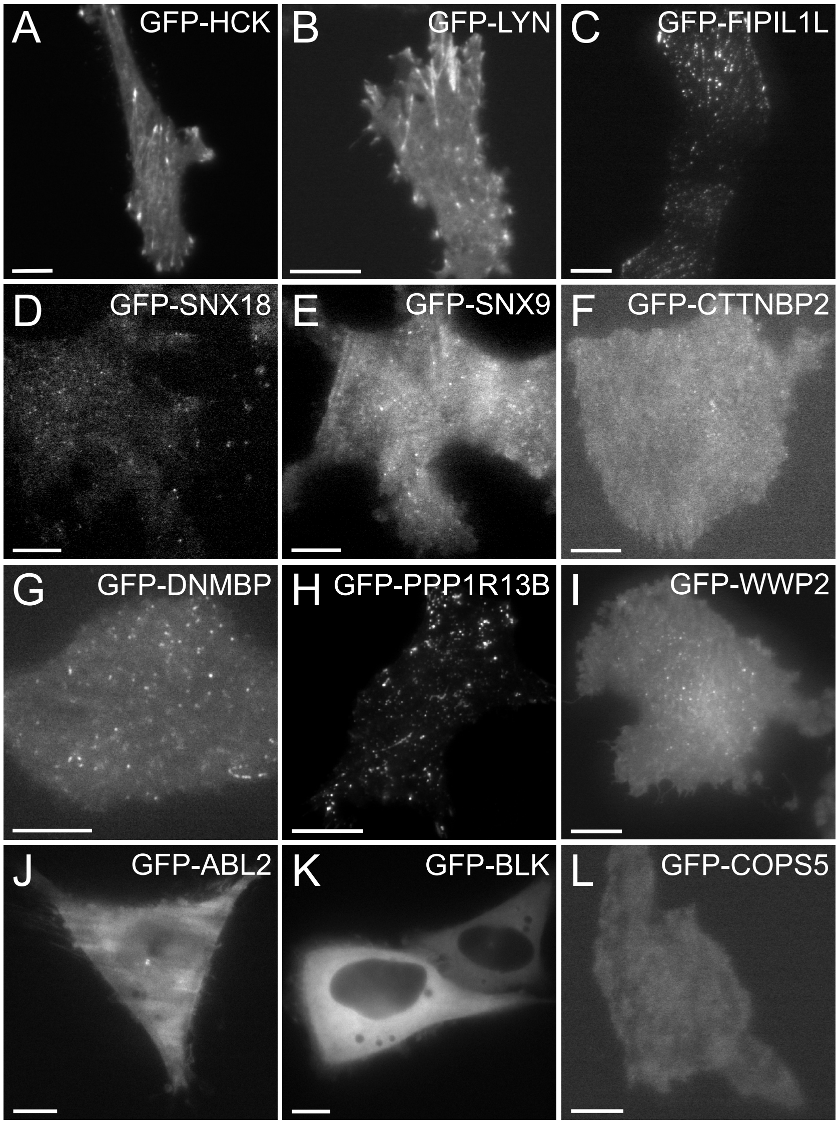

Supplementary Figure 9. Localization of GFP-tagged protein candidates. Localization of GFP-tagged protein candidates by epifluorescence or TIRF microscopy in human melanoma SK-MEL-2 cells. (A-C, H-L) Representative epifluorescence images of GFP-tagged HCK, LYN, FIPIL1L, PPP1R13B, WWP2, ABL2, BLK, and COPS5. (D-G) TIRF images of GFP-tagged SNX18, SNX9, CTTNBP2, and DNMBP. Scale bars, 10 μm. Order of image presentation corresponds to Supplementary Table 14, part 1 of 2.

Figure S10

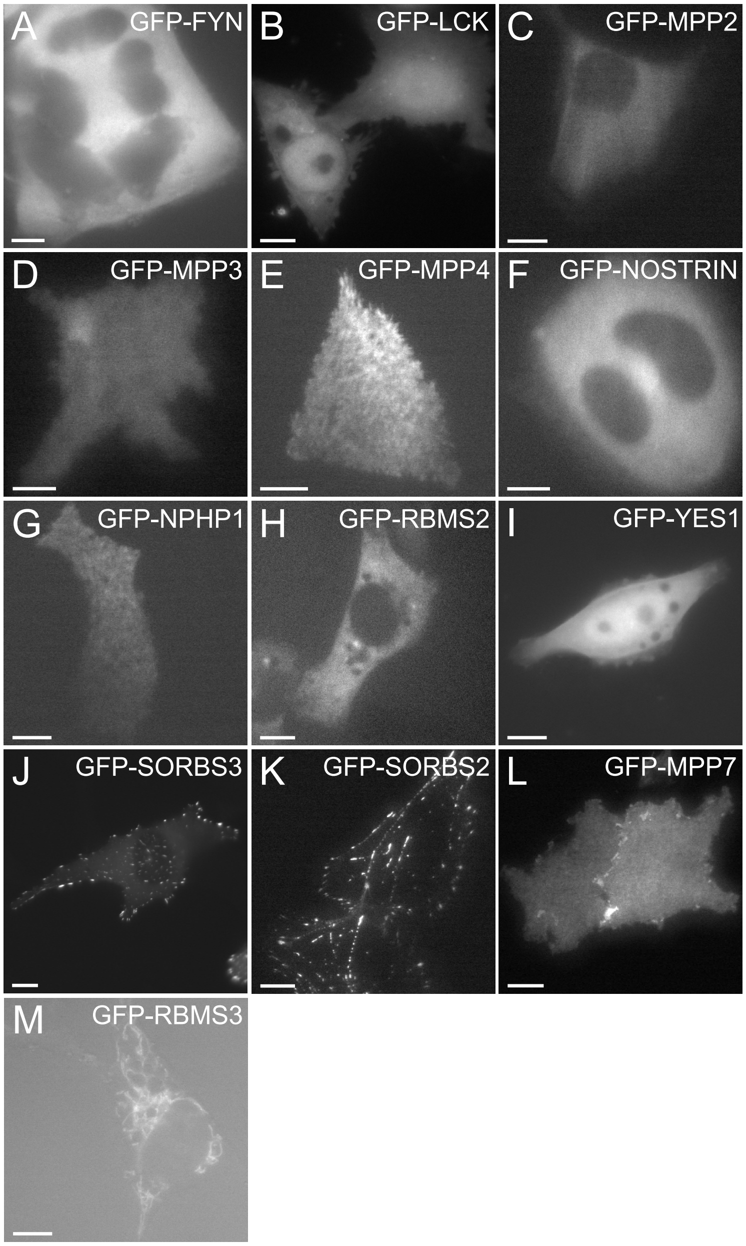

Supplementary Figure 10. Localization of GFP-tagged protein candidates. Localization of GFP-tagged protein candidates by epifluorescence microscopy in human melanoma SK-MEL-2 cells. (A-M) Representative images of GFP-tagged FYN, LCK, MPP2, MPP3, MPP4, NOSTRIN, NPHP1, RBMS2, YES1, SORBS3, SORBS2, MPP7, and RBMS3. Scale bars, 10 μm. Order of image presentation corresponds to Supplementary Table 14, part 2 of 2.

Figure S11

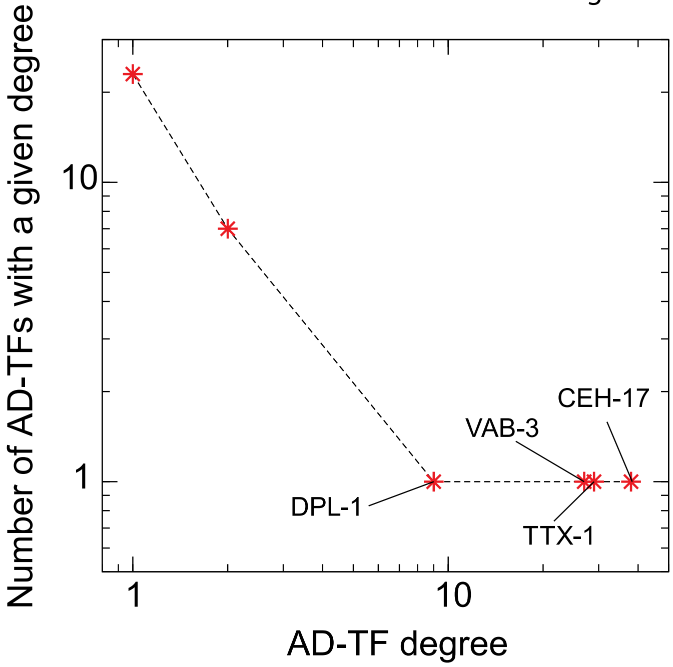

Supplementary Figure 11. Degree distribution of the AD transcription factors in our Y2H network. Four transcription factors (CEH-17, DPL-1, TTX-1 and VAB-3) were found to interact with a much larger number of SH3 domains than expected ($\geq$ 9) in Y2H screening. These are likely to be artifacts of the Y2H experiment and were removed from the final network.

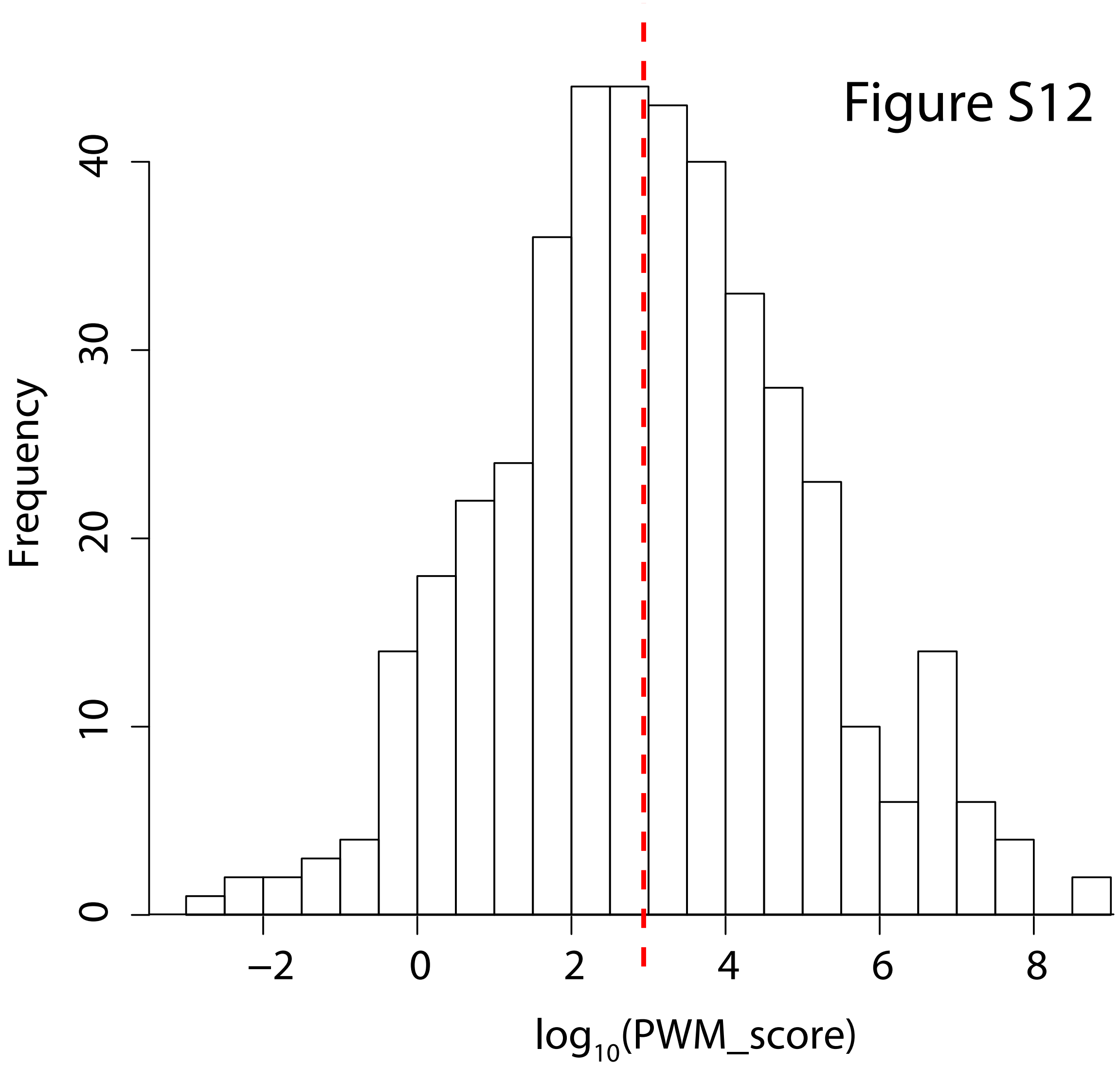

Supplementary Figure 12. Distribution of PWM scores among Y2H interactions. The distribution shows the score of SH3 mediated interactions supported by more than one line of evidence in the Y2H screen and for which the SH3 domain has a phage derived specificity profile. The red line indicates the position of the median (972), corresponding roughly to the threshold T=1000 taken on PWM scores to incorporate in the network Y2H interactions supported by only one colony and predict binding motifs on worm proteins.

Figure S13

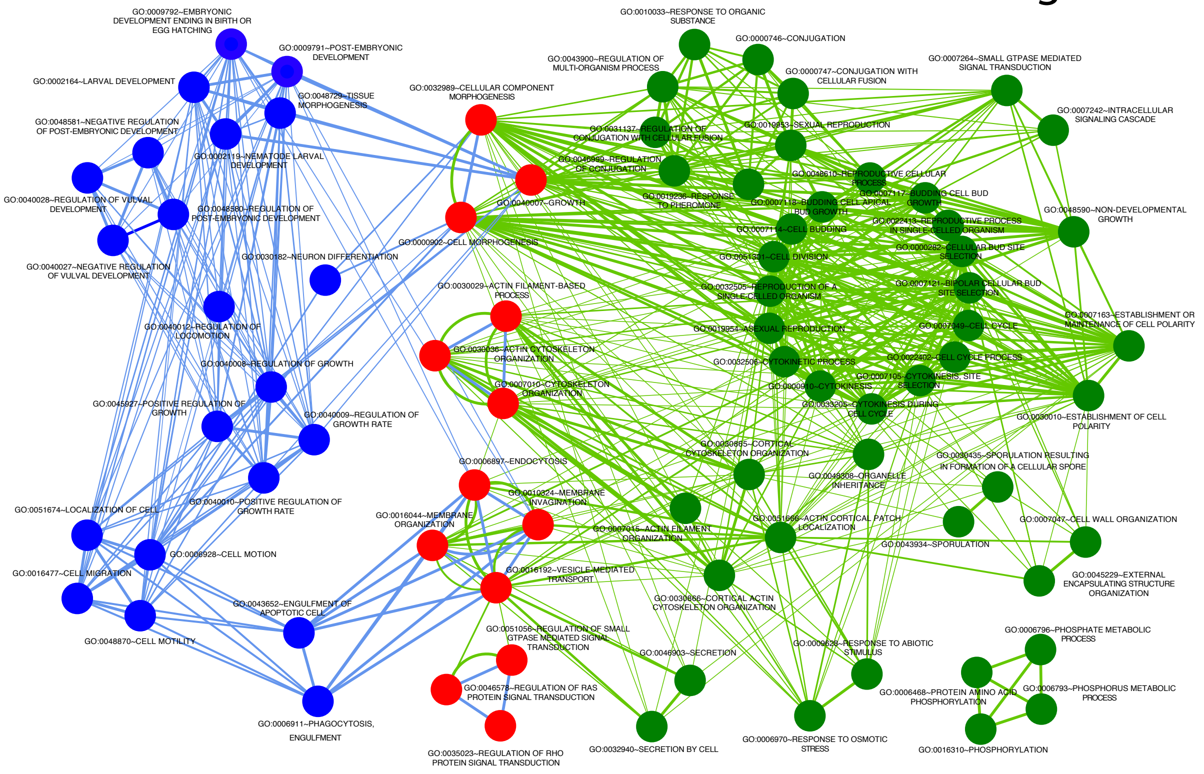

Supplementary Figure 13. Enrichment map of yeast and worm SH3 interactomes. This is the same figure as Figure 4 showing the label of each node. See caption for Figure 4.

3. Supplementary Tables

Supplementary Table 1. Worm SH3 domains used in phage display and yeast two-hybrid screens.

Supplementary Table 2. Hub baits used in yeast two-hybrid screens.

Bait ID	Sequence Name	Common Name	Range (amino acids)	Location from WorfDB
Hub01	C09G1.4	C09G1.4	full length	11001@G01
Hub02	C02C6.1	DYN-1	full length	11024@G07
Hub03	Y57G11C.24	EPS-8	full length	11051@F07
Hub04	F32D1.1	FIGL-1	full length	11001@A04
Hub05	F43B10.2	TAG-343	full length	11082@C10
Hub06	T17H7.4	GEI-16	full length	11010@G10
Hub07	T11B7.1	T11B7.1	full length	11049@B10
Hub08	T11B7.4	ALP-1	full length	11052@C02
Hub09	C08B11.5	SAP-49	full length	11085@H06
ITSN-1_EH	Y116A8C.36	ITSN-1	1-275	
ITSN-1_CC	Y116A8C.36	ITSN-1	269-663	
ITSN-1_SH3	Y116A8C.36	ITSN-1	657-1085	

Supplementary Table 3. Filtered yeast two-hybrid interaction list.

Supplementary Table 4. Overlap between the worm SH3 interactome and other datasets.

Reference networks	SH3 interactome bait proteins in reference	Interactions in reference mediated by a bait	Interactions in worm SH3 interactome mediated by baits also found in reference	Overlapping interactions	P-value (Fisher's exact test)
WI8	27	266	542	28	2.6×10^{-47}
Interologs	38	422	649	25	1.2×10^{-37}
WormNet	61	8923	893	51	1.2×10^{-28}

Supplementary Table 5. GO semantic similarity analysis.

Network	Number of PPIs (a)	Number of PPIs with a SIM score (b)	% of PPIs with a SIM score (c=b/a)	Sum of all SIM scores (d)	Average SIM score (d/b)	Overall Average SIM score (d/a)
Y2H	1070	150	14.00%	67.5	0.45	0.063
Random	1070	44	4.13%	18.3	0.43	0.017

Supplementary Table 6. List of SH3 domains whose protein is involved (or not) in known PPIs from iRefWeb database.

Supplementary Table 7. Worm SH3 interactome Gene Ontology Biological Process term enrichment.

Supplementary Table 8. Yeast SH3 interactome Gene Ontology Biological Process term enrichment.

Supplementary Table 9. List of rewiring events between Worm and Yeast. The first two columns show observed interactions between SH3 domains and other proteins, based on our worm SH3 interactome and the Yeast interactome of (Tonikian et al, 2009). Columns 3 and 4 show the orthologous proteins and proteins not found to interact. Column 5 shows the best conserved predicted binding site and Column 6 the best matching region in the ortholog protein. The binding site similarity (column 7) was computed as the percent sequence identity. The list of all predicted binding sites (*i.e.*, stretches of 15 amino acids with a PWM score $\geq$ T) is given in column 8, the best matching regions in the ortholog protein are given in column 9 and the corresponding similarities in column 10. In column 11, (i) indicates conserved binding site (% identity $\geq$ 0.5) and non-conserved SH3 specificity, (ii) indicates conserved specificity and non-

conserved binding site, (iii) indicates non-conserved specificity and non-conserved binding site. 0 stands for SH3 domains without phage display data in worm. Asterisks highlight interactions with a conserved SH3 specificity and a conserved binding site, although the interaction was experimentally detected in only one organism. Column 12 indicates whether the ortholog of the SH3-containing protein in column 1 (*i.e.*, the protein in column 3) also contains an SH3 domain that has been used as a bait in the corresponding SH3 interactome.

Supplementary Table 10. Curated worm endocytosis protein list.

Supplementary Table 11. List of yeast endocytosis proteins. The proteins were retrieved from Gene Ontology (GO:0006897) with experimental evidence codes (*i.e*, EXP, IDA, IPI, IMP, IGI, and IEP). Column 1 shows the protein name, column 2 shows the GO category (child of GO:0006897), columns 3 the evidence code and column 4 the literature references. Multiple evidences are separated by underscores.

Supplementary Table 12. Worm and human endocytosis protein predictions using the modified k-core algorithm with k = 3.

Supplementary Table 13. List of protein interactions among endocytosis proteins, grouped according to the different categories that are linked by these interactions (data used to build the thematic map of Supplementary Figure 6).

Supplementary Table 14. Cloned human ORFs used for validation of endocytosis protein predictions.

ENTREZ GENE ID	BC NUMBER	Symbol	K-core	ORF Length	Localization
3055	BC014435	HCK	13	1518	actin
4067	BC126456	LYN	13	1539	actin
9256	BC146852	BZRAP1	6	5574	actin stress fiber
55917	BC016029	CTTNBP2NL	9	1920	actin stress fiber

11259	BC027860	FILIP1L	9	2682	actin stress fiber
112574	BC117218, BC117220	SNX18	6	1887	CCP
51429	BC005022	SNX9	6	1788	CCP
4293	BC133706	MAP3K9	4	3315	CCP
152503	BC108890	SH3D19	9	2295	CCP
83992	BC106000	CTTNBP2	9	4992	cell surface puncta
23268	BC041628	DNMBP	11	2472	cell surface puncta
23368	BC136527	PPP1R13B	5	3273	cell surface puncta
11060	BC000108	WWP2	3	1008	cell surface puncta
27	BC065912	ABL2	9	3504	cytoplasmic
640	BC007371	BLK	13	1518	cytoplasmic
10987	BC001187, BC001859, BC007272	COPS5	5	1005	cytoplasmic
2534	BC032496	FYN	9	1449	cytoplasmic
3932	BC013200	LCK	13	1620	cytoplasmic
4355	BC030287	MPP2	4	1659	cytoplasmic
4356	BC056865	MPP3	4	1758	cytoplasmic
58538	BC132785	MPP4	4	1893	cytoplasmic
115677	BC014189	NOSTRIN	3	1287	cytoplasmic
4867	BC062574	NPHP1	5	1845	cytoplasmic
5939	BC027863	RBMS2	4	1224	cytoplasmic
7525	BC048960	YES1	9	1632	cytoplasmic
2268	BC064382	FGR	9	1590	focal adhesion
10174	BC067260	SORBS3	13	2016	focal adhesion
8470	BC011883	SORBS2	13	1938	focal adhesion, actin stress fiber
143098	BC038105	MPP7	4	1731	membrane ruffles
27303	BC117315	RBMS3	4	1302	tubular

Supplementary Table 15. List of proteins retrieved in the phospho-proteomics assay. Those matching predictions based on the worm SH3 interactome are shown at the top of the list.

Supplementary Table 16. List of competitive and coincident interactions. Interactions involving the same target protein are listed by pairs every two lines. Only interactions with a reliable predicted binding motif (PWM score > T) are considered.

Supplementary Table 17. List of peptides retrieved in the phage display experiments for each SH3 domain. Peptides binding to each domain have been manually aligned.

Supplementary Table 18. The different PWMs used to model the binding specificity of each SH3 domain.

Supplementary Table 19. List of ortholog relationships between worm and yeast proteins in the two SH3 interactomes.

4. References

Anggono V, Robinson PJ (2007) Syndapin I and endophilin I bind overlapping proline-rich regions of dynamin I: role in synaptic vesicle endocytosis. *J Neurochem* **102:** 931-943

Bjorklund AK, Light S, Sagit R, Elofsson A Nebulin: a study of protein repeat evolution. *J Mol Biol* **402:** 38-51

Breitkreutz BJ, Stark C, Reguly T, Boucher L, Breitkreutz A, Livstone M, Oughtred R, Lackner DH, Bahler J, Wood V, Dolinski K, Tyers M (2008) The BioGRID Interaction Database: 2008 update. *Nucleic Acids Res* **36:** D637-640

Harris BZ, Venkatasubrahmanyam S, Lim WA (2002) Coordinated folding and association of the LIN-2, -7 (L27) domain. An obligate heterodimerization involved in assembly of signaling and cell polarity complexes. *J Biol Chem* **277:** 34902-34908

Jin J, Xie X, Chen C, Park JG, Stark C, James DA, Olhovsky M, Linding R, Mao Y, Pawson T (2009) Eukaryotic protein domains as functional units of cellular evolution. *Sci Signal* **2:** ra76

Koonin EV, Aravind L, Kondrashov AS (2000) The impact of comparative genomics on our understanding of evolution. *Cell* **101:** 573-576

Letunic I, Doerks T, Bork P (2009) SMART 6: recent updates and new developments. *Nucleic Acids Res* **37:** D229-232

Merico D, Isserlin R, Stueker O, Emili A, Bader GD (2011) Enrichment map: a network-based method for gene-set enrichment visualization and interpretation. *PLoS ONE* **5:** e13984

Nakagawa H, Suzuki H, Machida S, Suzuki J, Ohashi K, Jin M, Miyamoto S, Terasaki AG (2009) Contribution of the LIM domain and nebulin-repeats to the interaction of Lasp-2 with actin filaments and focal adhesions. *PLoS One* **4:** e7530

Schultz J, Copley RR, Doerks T, Ponting CP, Bork P (2000) SMART: a web-based tool for the study of genetically mobile domains. *Nucleic Acids Res* **28:** 231-234

Shannon P, Markiel A, Ozier O, Baliga NS, Wang JT, Ramage D, Amin N, Schwikowski B, Ideker T (2003) Cytoscape: a software environment for integrated models of biomolecular interaction networks. *Genome Res* **13:** 2498-2504

Tonikian R, Xin X, Toret CP, Gfeller D, Landgraf C, Panni S, Paoluzi S, Castagnoli L, Currell B, Seshagiri S, Yu H, Winsor B, Vidal M, Davidson AR, Gerstein MB, Bader GD, Volkmer R, Cesareni G, Drubin DG, Kim PM et al (2009) Bayesian modeling of the yeast SH3 domain interactome predicts spatiotemporal dynamics of endocytosis proteins. *PLoS Biology* **7:** e1000218

Turner B, Razick S, Turinsky AL, Vlasblom J, Crowdy EK, Cho E, Morrison K, Donaldson IM, Wodak SJ (2010) iRefWeb: interactive analysis of consolidated protein interaction data and their supporting evidence. *Database (Oxford)* **2010:** baq023

Uhlik MT, Temple B, Bencharit S, Kimple AJ, Siderovski DP, Johnson GL (2005) Structural and evolutionary division of phosphotyrosine binding (PTB) domains. *J Mol Biol* **345:** 1-20

Zhang LV, King OD, Wong SL, Goldberg DS, Tong AH, Lesage G, Andrews B, Bussey H, Boone C, Roth FP (2005) Motifs, themes and thematic maps of an integrated Saccharomyces cerevisiae interaction network. *J Biol* **4:** 6
